# Microwaves assisted liver surgery in elderly patients: technical aspects of our first clinical experience

**DOI:** 10.1186/1471-2482-13-S1-A14

**Published:** 2013-09-16

**Authors:** Clemente De Rosa, Alberto Oldani, Manuela Monni, Alfonso Terrone, Marcello Garavoglia

**Affiliations:** 1 Department of Surgery, Clinical Surgery Division, Eastern Piedmont University , Azienda ospedaliero-universitaria “Maggiore della Carità” Novara, Italy

## Introduction

Surgical resection for liver malignancy, has been demonstrated to increase overall survival; although the rate of post-operative mortality was reduced from 20% to 4% in recent years, it remains in any case a procedure that requires experienced and dedicated surgeons so that it can be followed by satisfactory results [1].

Several techniques have been developed and proposed in recent years in order to reduce the bleeding during liver resection. Surgeons can use an low central venous pressure, the continuous or intermittent clamping of hepatic pedicle or total vascular exclusion.

The parenchymal section may instead be performed by different devices: the HF, dissector or ultrasonic scalpel, laser, stapler, radiofrequency energy. Radiofrequency ablation (RFA) is the most widely used modality for radical treatment of small HCC (_3 cm). It improves 5-year survival compared with standard chemotherapy and chemical ablation, allowing down-staging of unresectable hepatic masses. Microwave ablation (MWA) has been extensively applied in Asia and was recently introduced in the United States of America and Europe with excellent results, especially with regard to large unresectable HCC [2].

Microwave assisted surgery technology with the introduction of the latest technical expedient (“mini-choke”) has gained excellent therapeutic capability in comparison with RF assisted surgery.

Applying the concept of precoagulation of liver transection line we developed a new technique that provides the pre coagulation of the resection line using microwaves technologies [3].

The aim of this study is to evaluate the clinical feasibility and the results of liver resections in elderly patients performed using microwave energy to obtain blood-less surgery, rate of biliary leakege, speed of the procedure, and the onset of surgical complications.

## Methods

From June 2011 to April 2013, were subjected to surgical treatment thirteen patients older than seventy years including a male to female (M/F) ratio of 8:5.

Pathologies treated with resection were was hepatitis C virus (HCV)-related HCC (*n* _ 4; 30.8 %); hepatitis B virus (HBV)-related HCC (*n* _ 3; 23.1 %); gallbladder cancer (*n* _ 2; 15.4 %); liver metastases from colorectal cancer (*n* _4; 30.8 %). Surgical procedures included twelve minor liver resection (6 wedge resections, 4 segmentectomies, 2 subsegmentectomies). Only one case of laparotomic ablation treatment (HCC< 2cm) was perforrmed. The application of MW was performed under sonographic guidance and using Amica HS 14 Gauge needle with “mini-choke” technology. The operating frequency was 2450 MHz, power 20–80 W. Amica HS Antenna included a new device on the tip (“minichoke”) as a technical remedy to back heating effects, both due to the reflected waves and to ohmic dissipation along the feeding coaxial line (“comet-effect”). In all surgical procedures has been practiced the underpass of the hepatic hilum for the safe controlof bleeding.

## Results

The technique used for the parenchymal transection is similar to that previously described by our institute RF.assisted liver resection and applied only to minor resections. There was no need to perform occlusive clamping; the main vascular and biliary pedicles were sectioned between ligatures. The total operative time was 110 + / -50 min.

Post-operative blood transfusion has become necessary in only one patient, and in two cases of shearing liver hemostasis of parenchymal surface was completed by Argon-beam coagulator.

We haven’t found any cases of postoperative biliary leak; in three (23.1%) patients, non-surgical complications have arisen, and the mean hospital stay was 9 + /-7 days.

## Conclusions

Considerable progress has characterized the last years of preclinical and clinical research in the surgical management of primary and secondary liver neoplasm. Liver resections for malignancy in the elderly are characterized by the high rate of intra-and perioperative morbidity.

The improvement in surgical and anesthesiological techniques has made hepatectomy a safe procedure and has led many to consider as candidates to liver resection patients who only a few years ago, was denied the surgical option[4].

The meticulous haemostasis and an appropriate technique of parenchymal transection can limit these problems. MW energy is produced by electromagnetic fields with frequencies around 1 GHz. The radiation is applied via antennas stuck in the liver lesion under ultrasound guidance. A new microwave generator operating at frequencies of 2.45 GHz and equipped with an innovative device (“mini-choke”) has been developed to trapping in the tip energy that propagates in a retrograde fashion, responsible for the “comet-effect.” The presence of a water cooling system allows the antenna to avoid overheating due to heat dissipation along the line of microwave transmission. The introduction of the latest technological innovations (“mini-choke”) permits one to obtain a larger diameter figure of necrosis more quickly than with RF energy.

The figure of necrosis was characterized by complete reproducibility and did not suffer the limitations of inherent heat transfer by conduction or “heat-sink” effects due to proximity to the vascular structures.

In conclusion this study with a small group of patients suggest surgical advantages in terms of statement for best practice in resection of liver malignancy. It allows a complete resection obtaining a negative pathologic margin, no blood loss and need for blood transfusions factors predicting post operative morbidity and survival, and consistently reducing time of procedure and avoidance of liver ischemic injury. Further studies should confirm this preliminary data extending surgical indication to major hepatic resection [5].
